# The Role of Sirt3 in Kidney Health and Disease

**DOI:** 10.3390/ph18111668

**Published:** 2025-11-04

**Authors:** Ryan S. Azzouz, Liang-Jun Yan

**Affiliations:** Department of Pharmaceutical Sciences, UNT System College of Pharmacy, University of North Texas Health Science Center, Fort Worth, TX 76107, USA; ryanazzouz@my.unthsc.edu

**Keywords:** Sirt3, acute kidney injury, chronic kidney disease, oxidative stress, mitochondrial dysfunction, renal tubular cells

## Abstract

Sirtuin 3 (sirt3), a mitochondrial NAD^+^-dependent deacetylase, is an important enzyme in the maintenance of kidney functions, with critical roles in renal homeostasis, attenuation of oxidative stress, and preservation of mitochondrial homeostasis. This review aims to summarize the current literature on the mechanisms by which sirt3 impacts kidney health and disease, as well as highlight the therapeutic implications of sirt3 targeting. We conducted a PubMed search using the title word “sirt3” and the keyword “kidney” to generate our literature review sources. The animal studies that are explored in this review include cisplatin-induced acute kidney injury, cadmium-induced kidney injury, cecal ligation and puncture (CLP) and lipopolysaccharide-induced sepsis, diabetic kidney fibrosis, high-fat induced kidney disease, and ischemic kidney injury. Increasing evidence points towards a deficiency in sirt3 being an aggravator of mitochondrial dysfunction, promoting abnormal glycolysis, and contributing to the progression of diabetic kidney disease, renal fibrosis, and acute kidney injury. In contrast, pharmacological and dietary activation of sirt3 has been observed to enhance mitochondrial biogenesis, mitigate production of reactive oxygen species (ROS), and preserve the integrity of renal tubular cells under stressful conditions. Collectively, studies point towards sirt3 as a central metabolic and antioxidant regulator within the kidney, and link chronic kidney disease, as well as age-related decline in kidney function, to this enzyme. The conclusion of this review identifies future directions for translational research regarding sirt3 and NAD^+^-dependent regulation of mitochondrial homeostasis in renal medicine.

## 1. Introduction

The kidneys play an essential role in the maintenance of the body’s homeostasis through a diverse set of functions, including excretion of metabolic waste, regulation of fluid balance and blood pressure, acid–base equilibrium, and endocrine roles such as erythropoietin production and vitamin D activation [1,2,3,4]. In addition to the liver, the kidneys also contribute to systemic glucagon regulation of gluconeogenesis, especially under fasting conditions [5,6,7]. These functions occur through the kidneys’ complex filtration system, a highly ATP-dependent process that sees renal tubular cells relying on active transport processes to reabsorb solutes along the nephron. To meet the high energy demand, nephrons contain a high density of mitochondria and consume substantial amounts of oxygen [8]. This metabolic profile enables efficient solute handling and allows the kidney to fulfill its functions, but leaves renal tissue highly vulnerable to mitochondrial damage, oxidative stress, and metabolic reprogramming during injury [9,10].

Mitochondrial dysfunction has been recognized as a central mechanism in the pathogenesis of acute kidney injury (AKI) and chronic kidney disease (CKD) [11,12,13]. The literature surrounding these two conditions has shown that impaired oxidative phosphorylation, decreased fatty acid oxidation (FAO), and excess reactive oxygen species (ROS) production are all indicative of renal injury across models of ischemia-reperfusion, sepsis, toxin or cancer drug exposure, overnutrition, and diabetes [8,14,15,16,17]. One such toxin exposure study utilized the cancer drug cisplatin to demonstrate that mitochondrial fragmentation and impaired mitochondrial homeostasis could ensue due to exposure to this toxin [18]. Similarly, metabolic reprogramming is caused by abnormal glycolysis and suppression of FAO, both of which can drive fibrosis in diabetic kidney disease (DKD) [19,20]. Such discoveries further confirm that mitochondria can be both a sensor and amplifier for renal injury [21,22,23,24].

Sirtuins are a group of proteins that evolved to manage cellular stress responses [25]. They are NAD-dependent enzymes using NAD as their substrate to function as deacetylases or mono(ADP-ribosyl)transferases, thereby coordinating cellular response to a variety of stressors [25]. For example, both caloric restriction and exercise can activate sirtuins by elevating cellular NAD levels [26,27]. Certain sirtuins can also act as class III histone deacetylases and play an important role in epigenetics involving gene silencing, DNA recombination, and repair [28,29]. Sirt3, a mitochondrial NAD^+^-dependent deacetylase and a known regulator of mitochondrial metabolism [30,31,32], has been proven to act as a determinant of renal health [33,34,35,36]. Sirt3 expression has been observed in high quantities in metabolic tissues such as the kidney, where its role is to modulate the acetylation of mitochondrial proteins involved in the Krebs cycle, electron transport chain, and antioxidant defense [37]. It has been established that one of the mechanisms by which sirt3 is able to contribute to antioxidant defense is through the deacetylation and activation of manganese superoxide dismutase (SOD2), an important antioxidant enzyme whose expression levels are synergistically linked to sirt3 [38]. This example, along with others, shows sirt3’s ability to prevent oxidative injury and preserve mitochondrial integrity, thereby sustaining ATP generation.

Deficiencies or downregulation of sirt3 have been shown to contribute to the exacerbation of renal diseases [39,40,41,42]. In sepsis-induced AKI, it was found that knocking out the sirt3 gene would result in an increase in inflammasome activation, mitochondrial dysfunction, and tubular apoptosis [43]. Similarly, in a toxin exposure model using cisplatin to induce AKI, it was found that the presence of sirt3 resulted in amelioration of mitochondrial dysfunction, improved ATP production, and a mitigated ROS accumulation [44]. In ischemia-reperfusion models, sirt3 loss worsened kidney oxidative damage and renal fibrosis, while its restoration resulted in enhanced mitochondrial biogenesis and fusion [3].

Further evidence of sirt3’s important role in renal function can be observed in metabolic kidney disease, where it has been demonstrated that a deficiency in sirt3 can lead to abnormal glycolysis in diabetic kidneys with fibrosis. This highlights the shift away from FAO to maladaptive energy metabolism [20]. High-fat diet (HFD) models also demonstrate that an absence of sirt3 can accelerate lipotoxic mitochondrial injury and real fibrosis [45]. These studies establish sirt3’s role in the metabolic process as a protein capable of balancing oxidative metabolism against glycolysis and modulating cellular responses to stress.

On the other hand, therapeutic activation of sirt3 has been a growing area of interest [46,47,48], specifically into natural compounds such as silybin, resveratrol, honokiol, and melatonin. These compounds have shown promise in preclinical models in the enhancement of sirt3 expression and possible restoration of mitochondrial homeostasis [3,44,49,50]. These findings suggest that pharmacological modulation of sirt3 could provide a novel therapeutic approach for AKI and CKD. However, further mechanistic studies on these approaches must occur to optimize sirt3 targeting renal disease, before their real clinical applications can be explored.

This review aims to summarize current knowledge of sirt3 in kidney health and disease, with emphasis on its mechanistic roles in regulating metabolism, oxidative stress, apoptosis, and fibrosis. While many review articles exist in the literature regarding sirt3 and kidney disease [3,34,36,51,52,53], the novelty of our review is that we cover nearly all animal models of kidney injuries in the literature whereby sirt3 plays a clear role in preventing or mitigating renal dysfunction. Our review will also highlight therapeutic interventions aimed at activating sirt3 as a utility to mitigate the harm caused by AKI and CKD, with the latter including DKD. We then integrated findings across multiple experimental models of AKI, DKD, and CKD to provide a framework for understanding how modulation of sirt3 may offer new strategies for renal protection. It should be noted that this review will be limited to sirt3, as our laboratory is interested in studying the role of mitochondrial NAD-dependent redox enzymes in kidney disease. Nonetheless, this review is not meant to discount the roles of the other six sirtuin proteins in kidney disorders. For example, it has been well-established that sirt1, a cytosolic protein, also plays renoprotective roles in kidney injuries via delaying renal fibrogenesis, decreasing diabetic albuminuria, attenuating oxidative stress, and mitigating renal inflammation via NF-kB activation [54,55,56,57,58,59].

## 2. Sirt3 Structure and Function

**Figure 1:**
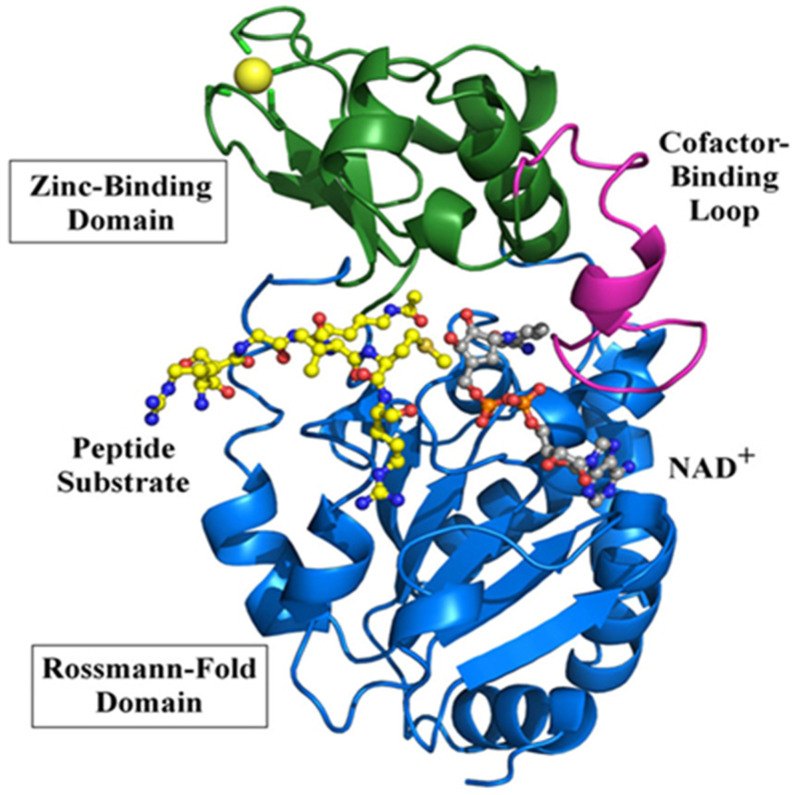
Three-dimensional structure of human sirt3 showing bound peptide (acetyl-CoA synthetase 2). NAD-binding Rossmann fold domain and a zinc-binding domain are also shown. Adapted from [69], Frontiers.org, 2012.

**Figure 2:**
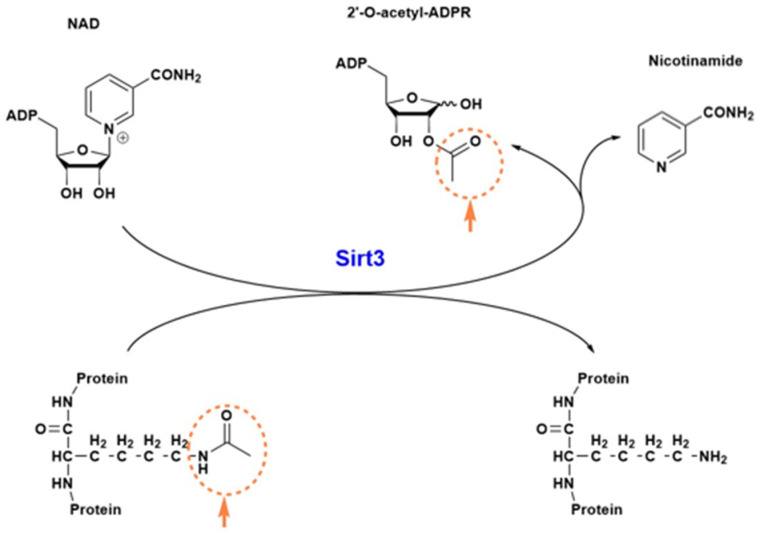
Sirt3 catalyzed deacetylation reaction. The acetyl group linked to a lysine residue (circled) is transferred to NAD^+^, which then splits into two molecules: nicotinamide and 2′-O-acetyl-ADPR. The deacetylated protein now restores its function.

In this section, we would like to have a brief discussion on sirt3’s structure and function. Sirt3 is one of the seven sirtuins [60] and is a family member of the class 3 histone deacetylase and is thus highly involved in epigenetic regulation [61]. It is a mitochondrial protein whereby its major function is to remove the acetyl functional group from numerous proteins that have undergone prior acetylation due to a variety of stress challenges [62]. Sirt3 uses NAD^+^ as its substrate when it catalyzes deacetylation and restores protein function. Given the role of NAD^+^ in energy metabolism, redox signaling, and cell death, sirt3 can exert its function via NAD^+^ utilization [52,63]. In addition to its NAD^+^ binding domain, sirt3 also has a zinc-binding domain and a peptide substrate binding pocket. It should be noted that sirt3 can readily undergo posttranslational oxidative modifications such as carbonylation [64,65], leading to loss of its ability to deacetylate and regulate other enzymes’ functions, such as SOD2 [66]. Such impairment can be counteracted by polyphenols and other natural antioxidants [66]. Figure 1 shows a three-dimensional structure of human sirt3, and Figure 2 shows sirt3’s catalyzed reaction of protein deacetylation whereby NAD^+^ is used to accept the acetyl group that is usually linked to a lysine residue [67,68].

As can be seen from this reaction, nicotinamide and 2′-O-acetyl-ADP-ribose (ADPR) are the co-products of the sirt3-catalyzed reaction. Removal of the lysine-linked acetyl group restores the protein function. Therefore, sirt3 is a regulatory enzyme that has been well-recognized for its role in redox signaling, anti-oxidative stress, anti-fibrosis, and anti-apoptosis [53].

## 3. Renoprotective Roles of Sirt3

### 3.1. Sirt3 Protects Against Cisplatin/Glycerol-Induced Acute Kidney Injury

Acute kidney injury is closely linked to mitochondrial dysfunction in renal tubular epithelial cells, where oxidative stress and mitochondrial damage can drive pathologies associated with this condition. Mitochondrial dysfunction that occurs because of AKI causes renal epithelial cells to lose their bioenergetic capacity and undergo apoptosis in response to injurious stimuli, such as toxins [3,8,70]. In widely used cisplatin and glycerol-induced models of AKI [71,72], sirt3 was demonstrated to be a central regulator of mitochondrial integrity. In these settings, sirt3 saw a marked reduction in its expression [18]. Treatment of mice with cisplatin resulted in mitochondrial fragmentation in the proximal tubules of the kidney, as well as a drop in the number of renal mitochondria in AKI mice when compared to controls. This was associated with increased accumulation of nitrotyrosine, a marker of protein oxidative damage [73], and decreased expression of PGC-1α, the main regulator of mitochondrial biogenesis [74]. In this model, both mRNA and protein expression of sirt3 dropped by nearly 80%, paralleled by decreased nicotinamide phosphoribosyltransferase (NAMPT), the rate-limiting enzyme for NAD^+^ biosynthesis.

In contrast, increasing sirt3 expression provided a protective feature against AKI. Treatment with AMPK agonist AICAR decreased blood urea nitrogen (BUN), reduced tubular necrosis, and preserved mitochondrial structure. This reversal was also associated with upregulation of NAMPT, PGC-1α, and, consequently, sirt3 in renal tissue [18]. Immunogold labeling revealed that cisplatin caused diffusion of mitochondrial proteins outside their predetermined areas. This diffusion could be reversed by AICAR treatment, which reinstated protein compartmentalization within mitochondria, suggesting that sirt3 aids in preserving organelle integrity. At the molecular level, sirt3’s protective effects extended to regulation of mitochondrial fission and fusion. In cisplatin-treated mice, dynamin-related protein 1 (DRP1) translocated to mitochondria, consistent with activation of fission pathways described previously in tubular injury [75]. AICAR treatment resulted in attenuated mitochondrial DRP1 accumulation, highlighting sirt3 as a restrainer of excessive fission and fragmentation [18].

Moreover, sirt3 deacetylase activity was assessed directly, using cisplatin-treated mice. In these mice, global mitochondrial protein acetylation increased, along with the loss of sirt3 function. AICAR restored deacetylase activity, reducing protein acetylation and reestablishing mitochondrial homeostasis [18,76]. Together, these findings show a mechanistic synergy between AMPK, NAMPT, PGC-1α, and sirt3 that preserves mitochondrial integrity and function when exposed to agents injurious to renal tissue. By counteracting oxidative damage, preventing uncontrolled fission, and sustaining mitochondrial integrity, sirt3 acts as a defender against AKI.

### 3.2. Sirt3 Protects Against Cadmium-Induced Kidney Injury

Cadmium is an environmentally heavy metal that can cause kidney injury upon acute or chronic exposure [77]. It can induce oxidative stress and mitochondrial dysfunction by promoting the acetylation of many proteins, including forkhead O3, SOD2, and glutathione peroxidase 4 [78,79], which is associated with a decreased expression of Sirt3 [78]. When resveratrol, a natural antioxidant, was administered under experimental conditions, sirt3 expression was enhanced and protein deacetylation was facilitated, leading to improved mitochondrial biogenesis and attenuated mitochondrial ROS metabolism [78]. Such studies also demonstrate that natural products such as resveratrol can activate the sirt3 signaling pathway to counteract heavy metal-induced renal toxicity [78,80].

### 3.3. Sirt3 Protects Against Sepsis-Induced AKI

Sepsis is one of the most common causes of acute kidney injury (AKI), with pathogenesis driven by inflammation, oxidative stress, and mitochondrial dysfunction in renal tubular epithelial cells (RTECs) [43,81]. Using a cecal ligation and puncture (CLP) model of sepsis, it has been demonstrated that sirt3 plays a protective role by attenuating changes in the mitochondria, limiting ROS production, and suppressing activation of NLRP3 inflammasome [43]. In wild-type (WT) mice subjected to CLP, BUN and serum creatine levels increased, along with tubular vacuolar degeneration, neutrophil infiltration, and mitochondrial damage. These changes were exacerbated in sirt3 knockout (KO) mice, where sirt3 deletion resulted in higher ROS levels, decreased mitochondrial density and volume, and worsened renal dysfunction [43].

Mechanistically, sirt3 protects against inflammasome-mediated injury. Mice that underwent CLP experienced upregulated NLRP3, ASC, and caspase-1, which drive expression of the proinflammatory cytokines IL-1β and IL-18. This response was enhanced in KO mice, confirming a protective anti-inflammatory role of sirt3 [13]. The results are backed up by reports that mark ROS as upstream activators of the NLRP3 inflammasomes, and that sirt3 mitigates this pathway by deacetylating and activating mitochondrial antioxidant enzymes such as SOD2 [38,82,83].

Sirt3’s role as a suppressor of ROS-driven oxidative injury was highlighted by Zhao et al. through a comparison to the known antioxidant N-acetylcysteine (NAC). Treatment with NAC partially restored BUN and serum creatinine levels in both WT and KO mice. NAC treatment also reduced tubular apoptosis and mitigated inflammatory interleukins and ROS production. This overlap between an antioxidant with known AKI-protective features of sirt3 suggests that sirt3 exerts its protective effect largely through suppression of the ROS-driven oxidative injury. In vitro experimentation confirmed this relationship [43]. Treatment of HK-2 cells with H_2_O_2_ increased ROS production, downregulated sirt3, and upregulated NLRP3. KO mice treated with H_2_O_2_ experienced significantly enhanced apoptosis, but their treatment with NAC partially restored cells to their normal function. Additionally, sirt3 overexpression inhibited H_2_O_2_-induced apoptosis, which suggests that sirt3 protects against sepsis-induced AKI via the ROS/Caspase pathway [43]. In a widely used lipopolysaccharide (LPS)-induced septic kidney injury model [84,85], sirt3 shows similar renoprotective effects [86,87] as observed in the CLP model.

Together, these findings highlight sirt3’s ability to act as a regulator of the mitochondrial redox balance and inflammatory signaling in septic AKI. Sirt3’s role in suppressing ROS, inhibiting NLRP3 activation, and limiting cytokine production allows it to preserve tubular integrity and renal function under septic stress.

### 3.4. Sirt3 Deficiency in Diabetic Kidney Disease

A decreased expression of sirt3 has been repeatedly observed in diabetic tissues [88,89,90,91]. In the kidney, renal fibrosis is the result of diabetic kidney disease (DKD) and stands as the leading cause of end-stage renal failure worldwide [92]. Kidney fibrosis is defined by features such as refraction of peritubular capillaries, accumulation of extracellular matrix (ECM) proteins, like collagen, expansion of activated myofibroblasts, and infiltration of inflammatory cells [93,94,95]. Although fibroblasts play a key role in the pathology of kidney fibrosis, their origin is not clearly defined. Researchers have suggested that the activation of myofibroblasts occurs because of the activation of resident fibroblasts and activation of mesenchymal transition programs in neighboring cells [20,95,96,97,98,99,100].

Sirt3 deficiency was proven to play a role in the development of kidney fibrosis in a streptozotocin (STZ)-induced diabetic CD-1 mouse model. Upon injection of these mice with STZ, sirt3’s expression was found to be markedly decreased [101,102,103]. The role of sirt3 in diabetic kidney disease was further explored by Srivastava et al., who demonstrated that sirt3 deficiency drives metabolic reprogramming in the diabetic kidney, pushing proximal tubular epithelial cells toward aerobic glycolysis as opposed to FAO [20]. In sirt3 KO mice with STZ-induced diabetes, renal cortical tissues experienced downregulation of PGC-1α, PPARα, CPT1a, and other FAO-related genes. This downregulation of FAO genes was coupled with the upregulation of the glycolytic enzymes hexokinase-2, pyruvate kinase M2 (PKM2), and pyruvate dehydrogenase kinase-1. Such changes in metabolic processes impaired mitochondrial respiration, elevated lactate production, and promoted ROS accumulation [20].

Notably, diabetic mice displayed an induction of PKM2 dimers, whereby dimerized PKM2 regulates HIF1α and IL-1β production and induces the activation of pro-glycolytic enzymes during inflammation and tumorigenesis, linking glycolysis directly to fibrosis [20,103,104,105,106]. These findings corroborate prior studies that defective FAO in renal tubular cells is a sufficient trigger to fibrogenesis and that HIF-1α-driven glycolysis promotes myofibroblast activation and ECM accumulation [107,108]. Mechanistically, sirt3 maintains mitochondrial integrity and redox balance through deacetylation of mitochondrial enzymes, which sustains FAO and represses HIF-1α-mediated transcription of glycolytic enzymes [20,107,108,109]. When sirt3 is absent, mitochondrial hyperacetylation, NAD^+^ depletion, and oxidative stress all occur, reinforcing glycolysis and fibroblast activation. Such evidence helps confirm that sirt3 is an important metabolic factor whose loss amplifies profibrotic metabolic signaling, making it a promising therapeutic target for diabetic kidney disease. It should be noted that the contents of sirt3 are robustly decreased in diabetic kidney injury, regardless of what animal models of DKD are used. Moreover, in terms of metformin and sirt3, it has been shown that metformin can activate AMPK, which in turn can decrease sirt3 SUMOylation via activating the cellular de-SUMOylation system, thereby leading to potential attenuation of kidney injury [110].

Table 1 shows the widely used rodent models of DKD for studying the role of sirt3 in diabetic nephropathy.

**Table 1:** Animal models that have been used for studying the role of sirt3 in DKD.

Diabetes Induction Method	References
High-fat diet (HFD)	[45,111,112]
Streptozotocin (STZ)	[20,89,113,114,115,116,117]
*db/db* mouse	[113,117,118]
STZ/HFD	[113,119]
STZ/High sugar/HFD	[120]
Zucker diabetic fatty rats	[121,122,123]

### 3.5. Sirt3 Protects Against High-Fat Diet-Induced Renal Injury

Metabolic syndrome, which includes clinical conditions such as obesity and hyperglycemia, is an established risk factor for chronic kidney disease [124,125,126]. Metabolic diseases have been shown to be associated with mitochondrial dysfunction that can be caused by post-translational lysine acetylation of mitochondrial proteins, a process that is in part controlled by sirt3 [127,128]. For example, sirt3 content can be downregulated by overnutrition, leading to diminished sirt3 capabilities of maintaining cellular homeostasis [83,129]. Likewise, in type 2 diabetic mouse models, it was found that mice that develop nephropathy because of hyperglycemia had decreased renal sirt3 mRNA expression and activity. This decrease in sirt3 function was associated with an increase in ROS levels and mitochondrial abnormalities [130].

Locatelli et al. demonstrated that sirt3-deficient mice exposed to high-fat diets developed more severe renal injury than wild-type controls [45]. Specifically, sirt3 deficiency led to earlier and more severe albuminuria than WT controls, beginning at four months of feeding compared to six months in WT mice. The progression of albuminuria was associated with podocyte dysfunction and glomerular capillary rarefaction [45]. Histological analysis showed increased mesangial matrix expansion, tubular vacuolization, and lipid accumulation in proximal tubules of sirt3^-/-^ mice when compared to WT mice that were treated by the same diet. Inflammatory cell infiltration, specifically that of the Mac-2-positive macrophages, saw an increase in the sirt3-deficient mice.

The increasingly severe renal injuries observed in sirt3^-/-^ mice were a result of elevated oxidative stress, mitochondrial abnormalities, and structural aberrations in tubular cells that occurred because of sirt3 being absent. This highlights sirt3’s enzymatic role in maintaining mitochondrial integrity during nutrient overload [45]. Sirt3 deficiency impaired the glomerular filtration barrier by causing podocyte cytoskeletal dysfunction, which was proven by decreased nestin expression, and by reducing glomerular endothelial density (CD31 expression). Taken together, these findings indicate that sirt3 can limit lipotoxicity-induced mitochondrial damage, inflammation, and podocyte injury in the setting of high-fat diets [45].

### 3.6. Sirt3 Protects Against Ischemia-Reperfusion-Induced Kidney Injury

Acute kidney injury is often incurred by ischemia-reperfusion injury [131,132,133,134], which can be induced by surgery, hemorrhage, and trauma [135]. It has been found that ischemic kidney injury is accompanied by increased acetylation of SOD2 and p53 [135] as well as early-stage renal fibrosis [136]. When sirt3 was inhibited by its selective inhibitors, such as 3-(1H-1,2,3-triazol-4-yl) pyridine (3-TYP), ischemic kidney injury was accentuated [137], indicating the involvement of sirt3 in ischemic injury. In contrast, when sirt3 was overexpressed, ischemic kidney injury was significantly mitigated. It was further found that the underlying mechanism was due to sirt3’s enhancement of mitochondrial fusion and activation of the ERK-OPA1 signaling pathway [137]. Sirt3 can also exert its renoprotective effects on ischemic kidney injury by modulating the DRP1 pathway involved in mitophagy [137]. Such studies collectively highlight the nephroprotective nature of sirt3 in ischemic kidney injury.

### 3.7. Dietary Restriction and Pharmacological Activation of Sirt3 in Renal Protection

Sirt3’s central role in protecting the kidneys from various deleterious conditions via maintenance of mitochondrial homeostasis and defending against oxidative stress leaves it as an ideal target of therapeutic strategies. In this section, we would like to discuss sirt3 activation by dietary restriction or caloric restriction and the pharmacological agents involved in renoprotection against kidney injuries.

#### 3.7.1. Dietary Restriction

Dietary restriction (DR), also known as caloric restriction (CR), is an established strategy for the retardation of aging and aging-associated diseases [138,139,140,141]. In the kidney, it has been demonstrated by Andrianova et al. that CR can upregulate sirt3 and ameliorate age-related kidney dysfunction [142]. The underlying mechanism is that sirt3 upregulation by CR mitigates oxidative stress as reflected by decreased levels of protein oxidation and lipid peroxidation. Moreover, mitochondrial membrane potential and mitochondrial integrity are also enhanced by CR [142]. The same research group also found that CR can confer ischemic tolerance to the kidney via sirt3 activation in senescence-accelerated rats [141]. CR achieves this mainly by sirt3-mediated enhancement of autophagy and mitophagy, leading to improvement in mitochondrial homeostasis and function [141]. It should be noted that fasting can also activate sirt3 for beneficial purposes [143,144]. It should also be noted that exercise can confer renoprotection against kidney injury by promoting mitochondrial dynamics via elevating the ATP/ADP ratio and NAD/NADH ratio, leading to AMPK/sirt3 activation and metabolic reprogramming and NF-kB inflammation suppression [144,145]. On the contrary, these mechanisms could not be activated in a sedentary lifestyle.

#### 3.7.2. Pharmacological Activation

Similar to CR, numerous pharmacological agents and natural products have been found to be able to activate sirt3, leading to amelioration of renal damage in a variety of animal models of kidney injury. For example, Yi et al. and Yang et al. have demonstrated that green tea polyphenols can attenuate kidney injury by a high-fat diet via activation of the ketogenic pathways and sirt3 [112,146]. Sun et al. reported that beta-nicotinamide mononucleotide alleviates septic acute kidney injury by activating the sirt3 signaling pathway [147]. Shi et al. reported that butyrate can attenuate high-fat diet-induced glomerulopathy via the sirt3 pathway [148]. Drugs such as dapagliflozin and roxadustat can also exert renoprotective effects via sirt3 activation [149]. Table 2 lists certain pharmacological agents and natural products that can activate the sirt3 signaling pathway and mitigate kidney injury in a variety of animal models of kidney disease.

**Table 2:** Pharmacological agents and natural products that can activate the sirt3 signaling pathway involved in renoprotection in a variety of animal models of kidney injury.

Pharmacological Agents/Natural Products	Kidney Injury Model	Main Mechanisms	References
Apelin	Cisplatin-AKI	Mitochondrial homeostasis	[150]
Kaempferol	High glucose-tubular cell damage	Decreased oxidative stress and apoptosis	[151]
Calycosin	A variety of models	Anti-oxidative stress	[152]
*S*-nitrosoglutathione	Septic kidney injury	Inhibition of pyroptosis	[153]
Sodium thiosulfate	Cisplatin-induced kidney injury	Increased H_2_S content	[154]
β-nicotiamide mononucleotide	Septic AKI	Increased NAD^+^ content	[147,155]
Artemisinin	Diclofenac-induced kidney injury	Mitochondrial homeostasis	[156]
Butyrate	High fat-induced glomerulopathy	Decreased oxidative stress	[148]
Luseogliflozin (SGLT2 inhibitor)	Ischemia-reperfusion injury	Suppression of ferroptosis	[157]
Ligustilide	Ischemia-reperfusion injury	Mitochondrial homeostasis	[158]
Tanshinone IIA	Doxorubicin-induced kidney injury	Decreased oxidative stress	[159]
Linagliptin	Cisplatin-induced kidney injury	Enhanced mitophagy	[160]
Dapagliflozin	Streptozotocin-DKD	Metabolic reprogramming	[117]
Dexmedetomidine	IFNα-induced glomerulopathy	Decreased inflammation and oxidative stress	[161]
Diosmin	UUO-mouse model	Decreased renal fibrosis	[162]
Dihydromyricetin	Gentamicin-induced nephropathy	Anti-oxidative stress	[163]
Salvianolic acid B	Diabetic nephropathy	Inhibiting oxidative stress	[120]
Dahuang-Gancao decoction	LPS-induced kidney injury	Decreased apoptosis/inflammation	[150]
Eprosartan	Ischemic-AKI	Decreased oxidative stress	[164]
Shilajit	5-fluorouracil kidney injury	Decreased oxidative stress	[165]
Dexpanthenol	Glycerol-induced kidney injury	Decreased oxidative stress	[166]
Nicotinamide riboside	db/db mice	Decreased inflammation	[167]
Canagliflozin	High salt renal injury	Decreased oxidative stress	[168]
Metrnl	DKD	Mitochondrial homeostasis	[169]
Maresin-1	Cecal ligation and puncture	Anti-inflammation	[170]
Notoginsenoside Fc	Acetaminophen-induced nephropathy	Decreased mitochondrial damage	[171]
Poricoic acid A	UUO rats	Suppressing renal fibrosis	[172]
TUG891/Fatty acid receptor 4	Ischemic/cisplatin/cecal ligation	Mitigating cell senescence	[173]
Magnesium isoglycyrrhizinate	Cisplatin-AKI	Decreased mito-DNA damage	[174]
Mitoquinone	Ischemia-reperfusion injury	Decreased oxidative damage	[175]
Molineria recurvata	Streptozotocin-DKD	Antioxidation and anti-inflammation	[115]
Eplerenone	Ischemia-reperfusion injury	Preserving mitochondrial function	[176]
Liquiritigenin	Cisplatin-induced AKI	Improved mitochondrial function	[177]
Matrine	Cisplatin-induced AKI	Enhanced mitochondrial function	[178]
Fluorofenidone	UUO/ischemic injury	Reduced mitochondrial damage	[179]
Honokiol	Cisplatin-AKI	Inhibiting mitochondrial fission	[180]
Purple rice husk	DKD	Maintaining redox balance	[181]
Melatonin	Contrast-induced AKI	Anti-oxidative stress	[182]
Melatonin	Cecal ligation & puncture	Decreased oxidative stress	[183]
Melatonin	Ischemic-AKI	Mitochondrial homeostasis	[184]
Tenovin-1	HFD-induced DKD	Antioxidant, anti-inflammation	[111]
N-acetylcysteine	Bisphenol A-induced renal injury	Restoring mitochondrial integrity	[185]
Mega-3 fatty acids	Rat 5/6 nephrectomy	Nrf2 activation	[186]
Apigenin	DKD	Restoring redox balance	[121]
Honokiol	DKD	Anti-oxidative stress	[130]
Rhein	5/6 nephrectomy-CKD	Anti-fibrosis & anti-oxidation	[187]
Empagliflozin	Streptozotocin-DKD	Inhibiting epithelial-mesenchymal transition	[188]
Jian-Pi-Yi-Shen formula	Adenine-induced CKD	Maintaining mitochondrial dynamics	[189]
Croton hookeri	Streptozotocin-DKD	Inhibiting oxidative stress	[114]
β-lapachone	Cisplatin-AKI	NQO1 activation	[190,191]
Spironolactone	5/6 nephrectomy	Increased p-eNOS production	[192]
Renalase	Cisplatin-AKI	Attenuating mitochondrial fission	[193]
Stanniocalcin-1	DKD	Inhibiting BNIP3	[194]
Metformin	Folic acid-AKI, ischemic AKI	Maintaining mitochondrial integrity	[110]
Intermedin	5/6 nephrectomy	Inhibiting oxidative stress	[195]
Pyrroloquinoline quinine	High glucose/HK-2 cells	Inhibiting ROS production	[196]
Mito-TEMPO	5/6 nephrectomy	Mitigating renal fibrosis	[197]
Fenofibrate	Salt-induced hypertension	Mitochondrial homeostasis	[198]
Dioscin	Fructose-induced renal injury	Decreased oxidative stress	[199]
Green tea polyphenols	High fat-induced renal damage	Decreased oxidative stress	[112]
*Lactobacillus rhamnosus* GG	Deoxynivalenol-induced kidney injury	Decreased oxidative damage	[200]
Annexin A1	Ischemic-AKI	Increased mitochondrial function	[201]
Jinshuiqing	IgA nephropathy	Anti-inflammation	[202]
Tert-butylhydroquinone	Contrast-induced nephropathy	Increased antioxidant capacity	[203]
Curcumin	Cisplatin-AKI	Mitochondrial homeostasis	[204,205]
Silybin	Cisplatin-AKI	Attenuated mitochondrial dysfunction	[44]
Resveratrol	Cadmium-induced renal damage	Maintaining redox balance	[78]
Resveratrol/exercise	Salt-induced hypertension	Enhanced mitophagy	[206]
ACE inhibitor/nicorandil	CKD	Decreased oxidative stress	[207]
Coumarin derivative SZC-6	CKD	Restoring mitochondrial function	[208]

Please note that this table is not meant to be exhaustive. Abbreviations: AKI, acute kidney injury; DKD, diabetic kidney disease; CKD, chronic kidney disease; HFD, high-fat diet; LPS, lipopolysaccharide; UUO, unilateral ureteral obstruction.

The studies shown in Table 2 underline the ability of sirt3 to be a suitable therapeutic target for renal disease and demonstrate various dietary supplements and natural compounds as effective sirt3 activators. Through upregulation of sirt3, these activators have shown the ability to restore mitochondrial homeostasis and enhance the kidney’s antioxidant defense. Through these actions, they may provide an avenue for preventing kidney injury in clinical settings. It should be noted that those plant compounds shown in Table 2 may only play a modulating role involving sirt3 activation. It should also be noted that many of the agents/mechanisms that activate sirt3 could also activate other sirtuins. Therefore, sirt3 activation shown in Table 1 may lack specificity, which needs to be addressed in future studies. Finally, given that sirt3 plays its renoprotective roles through protein deacetylation, as shown in Figure 2, Table 3 lists some of the well-studied sirt3 substrates involved in kidney function and dysfunction.

**Table 3:** Substrates that can be deacetylated by sirt3 in kidney disease.

Substrate Protein	Disease Model	Reference
Mitochondrial pyruvate carrier 2	DKD	[68]
Glycogen synthase kinase-3β	UUO	[42]
Carnitine palmitoyltransferase 1α	DKD	[209]
P53	Adenine-induced CKD	[47]
Krüppel-like factor 15	DKD	[210]
Transcription factor A	LPS-induced sepsis	[211]
Mitochondrial SOD2	Folic acid/ischemia model	[110]
Beta-catenin (lysine 49)	UUO	[172]
ATP-dependent metalloprotease (YME1L1)	LPS-AKI	[87]
Optic atrophy 1	Cisplatin-AKI	[178]
Pyruvate dehydrogenase E1α	UUO	[212]
Peroxisome proliferator-activated receptor gamma coactivator-1 alpha	5/6 Nephrectomy	[186]
ATP synthase β	Cisplatin-AKI	[213]
Forkhead box protein O3a	Hypertensive renal injury	[214]

## 4. Conclusions

**Figure 3:**
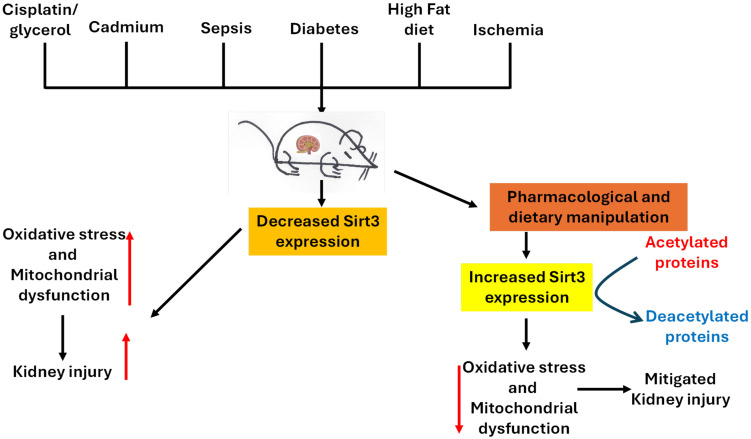
A schematic diagram showing animal models and sirt3’s renal protective mechanisms discussed in the article. Sirt3 can be activated by diet manipulation and pharmacological agents or natural products to enhance mitochondrial function by mitigating oxidative stress, renal fibrosis, and inflammation in a variety of kidney injuries.

**Figure 4:**
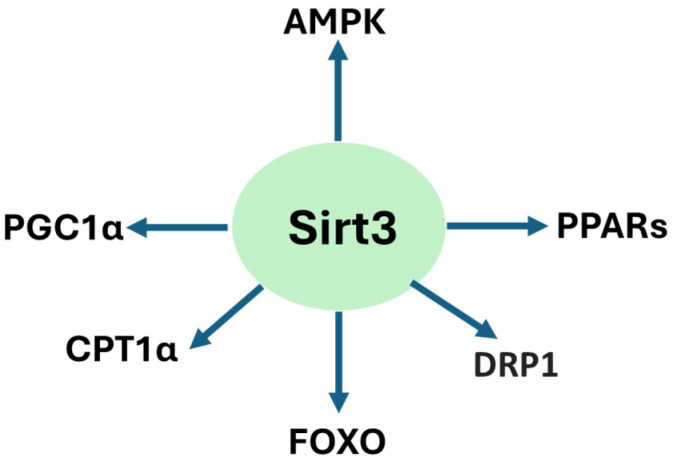
Sirt3-activated signaling pathways involved in renoprotection discussed in the review.

Sirt3 is a pivotal enzyme in renal mitochondrial regulation, conferring protective mechanisms by maintaining oxidative metabolism, suppressing ROS production, and preserving mitochondrial integrity [3,18,38]. Across models of acute kidney injury, ischemic kidney injury, diabetic kidney disease, and nephropathy induced by drugs, heavy metals, and high-fat diet, sirt3 deficiency has been noted to aggravate mitochondrial damage, promote abnormal glycolysis, and accelerate fibrosis [20,43,45]. Conversely, sirt3 upregulation or pharmacological stimulation reinstates mitochondrial homeostasis, preserves fatty acid oxidation, and inhibits inflammatory and apoptotic pathways in the kidney [18,44,130,215,216]. It should be noted that sirt3 could also be activated to enhance cancer cell adaptation and survival. Therefore, pharmacological inhibition of sirt3 activity could promote cancer cell death, thereby benefiting kidney health [217]. Figure 3 summarizes animal models of kidney injury and sirt3’s renal protective mechanisms discussed in this review and Figure 4 shows the signaling pathways that can be activated by sirt3 discussed in the review.

As a highlight, natural compounds such as silybin, resveratrol, honokiol, and melatonin have demonstrated their abilities to enhance sirt3 activity and elicit renoprotection in preclinical models [44,211,218]. While these findings establish sirt3 as an appealing therapeutic target, more studies will be needed to clarify their tissue-specific mechanisms, evaluate long-term modulation strategies, and translate these results into safe, effective therapies for use in clinics [3,219].

Taken together, current evidence positions sirt3 both as a biomarker of renal metabolic health and as a primary target for the development of new medications. The future of research integrating mechanistic insight with translational intent will be decisive for realizing sirt3’s potential to prevent or reverse kidney injury in human disease. Given that sirt3 competes with other NAD^+^-utilizing enzymes such as NAD kinase [220,221], CD38 [119,222], and dihydrolipoamide dehydrogenase [223,224], as well as other alpha-keto acid dehydrogenase complexes [225,226], functional characterization of these other NAD^+^-dependent enzymes and their relationship to sirt3 would also need to be investigated in a variety of kidney diseases.

## Data Availability

Data will be made available on request.
